# Discovery of new angiotensin converting enzyme (ACE) inhibitors from medicinal plants to treat hypertension using an *in vitro* assay

**DOI:** 10.1186/2008-2231-21-74

**Published:** 2013-12-20

**Authors:** Niusha Sharifi, Effat Souri, Seyed Ali Ziai, Gholamreza Amin, Massoud Amanlou

**Affiliations:** 1 Department of Medicinal Chemistry, Faculty of Pharmacy, Tehran University of Medical Sciences, Tehran, Iran; 2 Department of Pharmacology, Faculty of Medicine, Shahid Beheshti University of Medical Sciences, Tehran, Iran; 3 Department of Pharmacognosy, Faculty of Pharmacy, Tehran University of Medical Sciences, Tehran, Iran; 4 Medicinal Plants Research Center, Faculty of Pharmacy, Tehran University of Medical Sciences, Tehran, Iran

**Keywords:** Angiotensin converting enzyme, Screening, Medicinal plants, Total phenolic content, Antioxidant activity

## Abstract

**Background and purpose of the study:**

Angiotensin converting enzyme (ACE) inhibitors plays a critical role in treating hypertension. The purpose of the present investigation was to evaluate ACE inhibition activity of 50 Iranian medicinal plants using an *in vitro* assay.

**Methods:**

The ACE activity was evaluated by determining the hydrolysis rate of substrate, hippuryl-L-histidyl-L-leucine (HHL), using reverse phase high performance liquid chromatography (RP-HPLC). Total phenolic content and antioxidant activity were determined by Folin-Ciocalteu colorimetric method and DPPH radical scavenging assay respectively.

**Results:**

Six extracts revealed > 50% ACE inhibition activity at 330 μg/ml concentration. They were *Berberis integerrima* Bunge*.* (Berberidaceae) (88.2 ± 1.7%), *Crataegus microphylla* C. Koch (Rosaceae) (80.9 ± 1.3%), *Nymphaea alba* L. (Nymphaeaceae) (66.3 ± 1.2%), *Onopordon acanthium* L. (Asteraceae) (80.2 ± 2.0%), *Quercus infectoria* G. Olivier*.* (Fagaceae) (93.9 ± 2.5%) and *Rubus* sp. (Rosaceae) (51.3 ± 1.0%). *Q. infectoria* possessed the highest total phenolic content with 7410 ± 101 mg gallic acid/100 g dry plant. Antioxidant activity of *Q. infectoria* (IC_50_ value 1.7 ± 0.03 μg/ml) was more than that of BHT (IC_50_ value of 10.3 ± 0.15 μg/ml) and Trolox (IC_50_ value of 3.2 ± 0.06 μg/ml) as the positive controls.

**Conclusions:**

In this study, we introduced six medicinal plants with ACE inhibition activity. Despite the high ACE inhibition and antioxidant activity of *Q. infectoria*, due to its tannin content (tannins interfere in ACE activity), another plant, *O. acanthium,* which also had high ACE inhibition and antioxidant activity, but contained no tannin, could be utilized in further studies for isolation of active compounds.

## Introduction

In 2000, 26.4% of the world’s population suffered hypertension and it is predicted that this rate would increase by 60% in 2025 [1]. Since the proportion of hypertensive people will increase rapidly, new therapeutic approaches for management of hypertension are essential. High blood pressure is a silent killer, causing several serious diseases such as heart failure, kidney failure and stroke. There are a number of choices for the treatment of hypertension. Some treatments include diuretics, β-blockers, calcium channel blockers and angiotensin II receptor blockers, the most common of which is angiotensin converting enzyme inhibitors.

Angiotensin converting enzyme, EC 3.4.15.1, is a zinc metallopeptidase that converts the angiotensin I (inactive decapeptide) to angiotensin II (a potent vasoconstrictor), and bradykinin (a hypotensive peptide) to inactive components [2]. High ACE activity leads to increased concentration of angiotensin II and hypertension. Therefore, development of agents that inhibit the conversion of angiotensin I to angiotensin II, and bradykinin to inactive components began as a therapeutic strategy to treat hypertension. ACE inhibitors such as captopril and lisinopril play key roles in treating hypertension and maintaining the electrolyte balance [3]. They are commonly used as they are safe and well tolerated with few side effects.

Tannins are plant polyphenolic compounds that precipitate proteins and interfere in the functions of many macromolecules including ACE. Therefore, plants with ≥ 50% ACE inhibition activity would be further tested for the presence of tannins in order to eliminate false positives [4].

Furthermore, reactive oxygen species (ROS) play a significant role in cardiovascular diseases such as hypertension and congestive heart failure. In hypertensive patients, angiotensin II increases chronically and nicotinamide adenine dinucleotide phosphate (NADPH) oxidase is activated, which causes a rise in ROS. As a result, it is more beneficial for an antihypertensive drug to have antioxidant effect [5]. Phenolic compounds have antioxidant activity and are effective agents to prevent oxidative stress.

Natural products could be important sources of ACE inhibitors such as captopril, a synthetic antihypertensive drug, which is developed by changing and optimizing the structure of the venom of the Brazilian viper. Active substances derived from medicinal plants can also be a source of new ACE inhibitors. Moreover, some plants contain a great amount of phenolic compounds, so consequently, they have antioxidant activity.

In this study, some medicinal plants that are used to manage different diseases were screened to discover possible new ACE inhibition activity using an *in vitro* ACE inhibition assay. Among the plants tested, the most active ones were examined for total phenolic content and antioxidant activity.

## Material and methods

### Chemicals

Angiotensin converting enzyme (ACE) from rabbit lung, hippuryl-L-histidyl-L-leucine (HHL), 4-(2-hydroxyethyl)-1-piperazineethanesulfonic acid (HEPES) buffer, hippuric acid (HA) and captopril were purchased from Sigma-Aldrich Co. (England). HCl, KH_2_PO_4_, methanol (HPLC grade), 2,2-diphenyl-1-picrylhydrazyl (DPPH), Folin-Ciocalteu reagent, Na_2_CO_3_, gallic acid, butylated hydroxyl toluene (BHT), 6-hydroxy-2,5,7,8-tetramethylchroman-2-carboxylic acid (Trolox), FeCl_3_, NaCl, NaOH and dimethyl sulfoxide (DMSO) were purchased from Merck Co. (Germany). Ultrapure water was applied to prepare all of the aqueous solutions.

### Apparatus

Enzymatic incubation was performed in a thermomixer eppendorf comfort (Germany). HPLC analysis was carried out by a Knauer liquid chromatograph, with an ODS Eurospher column (250 × 4.6 mm, 100–5; C18), protected by a C18 precolumn (Perfectsil Target, ODS-3 (5 μm)) and a 20 μl injection loop. A smartline Photodiode Array (PDA) detector 2850 (Knauer, Germany) was used to detect analytes, and a Chromgate software version 3.3, was used for data processing. A Cecil UV/Vis spectrophotometer (series 9000) was utilized to measure the absorbances.

### Plant materials

Some of the studied plants (41 plants) were purchased from a local herbal store located in Tehran, Iran (June 2011). *Rubus* sp*.* was collected from north of Iran, Mazandaran province (June 2012), and 8 other plants were collected from Herburatum of Faculty of Pharmacy, Tehran University of Medical Sciences (June 2011). All of the mentioned plants were identified by Prof. G. Amin. Voucher specimens of the collected plants were deposited in the Herbarium of Tehran University of Medical Sciences.

### Preparation of crude extracts

Dried plant materials (1 g) were extracted with 20 ml methanol:water (80:20, v/v) at room temperature for 24 h and then over 2 h in an ultrasonic bath [6]. The extracts were filtered and concentrated under reduced pressure, using a rotary evaporator at room temperature and then they were lyophilized.

### ACE inhibition assay

In this study, the assay method is based on the hydrolysis of the substrate HHL by ACE, and measuring the amount of HA using RP-HPLC [6-9]. HEPES buffer solution used in this assay was prepared by dissolving 50 mM HEPES and 300 mM NaCl in 1000 ml water and adjusting the solution to pH 8.3 by 1 M NaOH solution. The substrate solution (9 mM) was prepared by dissolving HHL (19.74 mg) in 5 ml of HEPES buffer. Herbal extract (1 mg) was dissolved in 1 ml of solvent containing buffer/DMSO (90:10, v/v) to provide 330 μg/ml concentration (a comparable scale all over the world) [10].

First, ACE solution (25 μl) (80 mU/ml) was added to 25 μl of inhibitor solution (or solvent as negative control). After 3 min preincubation at 37°C, 25 μl substrate solution was added and the mixture was incubated at 37°C for 30 min with shaking at 300 rpm in an Eppendorf thermomixer. After 30 min, the reaction was stopped by addition of 50 μl of 1 M HCl and then the reaction mixture was subjected to RP-HPLC. The mobile phase was an isocratic system consisting of a mixture of 10 mM KH_2_PO_4_ (adjusted to pH 3 with H_3_PO_4_) and methanol (50:50, v/v). The flow rate was 1 ml/min and the injection volume was 20 μl. Analytes were detected by a PDA detector at the wavelength of 228 nm.

### ACE inhibition measurement

ACE inhibition calculation was based on the ratio of the area under curve (AUC) of HA peak in an inhibitor sample to that of negative control sample as it is expressed by equation 1:

(1) $$\begin{array}{c} \mathrm{ACE}\;\text{inhibition}\;\left(\%\right)=\left[1‒\left(\text{AUC}{\;}_{\text{inhibitor}}/\text{AUC}{\;}_{\text{control}}\right)\right]\;\\ \;\times \;100\;\\ \text{AUC}{\;}_{\text{inhibitor}}:\;\text{AUC}\;\text{of}\;\text{the}\;\text{HA}\;\text{peak}\;\text{with}\;\text{inhibitor}\\ \text{AUC}{\;}_{\text{control}}:\;\text{AUC}\;\text{of}\;\text{the}\;\text{HA}\;\text{peak}\;\text{of}\;\text{control}\;\text{sample}\;\text{without}\;\text{inhibitor} \end{array}$$

### Gelatin salt block test (eliminating false positive results)

In order to detect tannins, extracts with ≥ 50% ACE inhibition activity were tested under gelatin salt block test. Active extracts (200 mg) were dissolved in 4 ml water (50°C) and allowed to reach room temperature. To remove non-tannin compounds, a few drops of an aqueous 10% NaCl solution was added to the extracts. After filtration, the filtrate (1 ml) was added to four test tubes. Tube 1 contained 4–5 drops of 1% gelatin solution. Tube 2 contained 4–5 drops of 1% gelatin + 10% NaCl solution. Tube 3 contained 3–4 drops of 10% ferric chloride solution, and tube 4, was a control sample containing no reagent. Tube 1 and 2 were observed for the formation of precipitate, while tube 3 was observed for color [4].

The presence of greenish-blue or greenish-black color following the addition of ferric chloride indicates the presence of condensed tannins, and bluish-black color accounts for the presence of pyrogallol type tannins (assuming that precipitation is the result of gelatin salt block test). The presence of greenish-black or bluish-black color after the addition of ferric chloride with negative gelatin salt block test indicated no tannins in that extract.

### Total phenolic content (TP)

Folin-Ciocalteu colorimetric method [11] was used to determine the total phenolic content of extracts. First, Folin-Ciocalteu reagent (0.5 ml) was added to 0.5 ml of methanol extract solution (100 mg/ml). Then Na_2_CO_3_ (0.5 ml) (100 mg/ml) was added to the mixture. The absorbance was measured at 760 nm, after 60 min incubation at room temperature. Calibration curve of gallic acid in different concentrations (1, 10, 100, 500 and 1000 μg/ml) was prepared using the same method. Total phenolic content of each extract was calculated from calibration curve of gallic acid and reported as mg of gallic acid equivalent in 100 g dry of plant [12].

### DPPH radical scavenging activity

The DPPH radical scavenging activity assay is a method for determining the ability of extracts to trap free radicals [13]. First, DPPH methanol solution (2.0 ml) (0.16 mM) was added to 2.0 ml of extract methanol solutions in different concentrations. Next the same sample was prepared with 2.0 ml methanol replacing extract methanol solution as control. After that the mixtures were vortexed for 1 min and left at room temperature for 30 min. Later the absorbance (Abs) were read at 517 nm. For calculating radical scavenging activity (RSA), the equation 2 was used:

(2) $$\begin{array}{l} \mathrm{RSA}\;\left(\%\right)\;=\;\left[1‒\;\left(\mathrm{Abs}{\;}_{\mathrm{extract}}/\mathrm{Abs}{\;}_{\mathrm{control}}\right)\right]\times 100\\ \mathrm{Abs}{\;}_{\mathrm{extract}}:\;\mathrm{Absorbance}\;\mathrm{of}\;\mathrm{the}\;\mathrm{extract}\;\mathrm{sample}\\ \mathrm{Abs}{\;}_{\mathrm{control}}:\;\mathrm{Absorbance}\;\mathrm{of}\;\mathrm{the}\;\mathrm{control}\;\mathrm{sample} \end{array}$$

Radical scavenging activity of extracts was compared with those of BHT and Trolox as the positive controls.

### Establishing the sensitivity of the ACE assay system

The half maximal inhibitory concentration (IC_50_) value of captopril was determined from concentration - response curve by nonlinear regression, using GraphPad Prism software version 5, and it was compared with the value in the literature. The IC_50_ value for captopril determined in this study was 25 ± 2.6 nM, which was close to the value (23 nM) in the literature [14].

## Results

### ACE inhibition activity

Table 1 illustrates scientific names, plant families, common names in Persian, parts used, collection sites, collection times, medicinal uses, voucher numbers and ACE inhibition activities of herbal extracts. Of a total of fifty herbal extracts, six extracts have shown inhibition activity more than 50% at 330 μg/ml concentration (Table 2). They were *B. integerrima* (88.2 ± 1.7%), *C. microphylla* (80.9 ± 1.3%), *N. alba* (66.3 ± 1.2%), *O. acanthium* (80.2 ± 2.0%), *Q. infectoria* (93.9 ± 2.5%) and *Rubus* sp. (51.3 ± 1.0%).

**Table 1:** Angiotensin converting enzyme (ACE) inhibition activity of the 50 studied plants*

**Scientific name and plant family**	**Common name in Persian**	**Part used**	**Collection site**	**Medicinal use**	**Voucher No**	**Inhibition%**
*Abrus precatorius* L. (Leguminosae)	Cheshm- e Khorus	Seed	LHS^a^	Tonic, astringent	PMP - 725^b^	-13.1
*Alcea* sp. (Malvaceae)	Khatmi	Flower	LHS	Diuretic, demulcent, disinfectant	PMP - 503	-27.3
*Allium cepa* L. (Alliaceae)	Tokhm- e pyaz	Seed	LHS	Anti-typhoid fever	PMP - 726	21.2
*Amaranthus lividus* L. (Amaranthaceae)	Tokhm- e tajkhorus	Seed	LHS	Treatment of anemia	PMP - 723	-13.9
*Amomum subulatum* Roxb. (Zingiberaceae)	Hel- e bad	Fruit	LHS	Stomach tonic, carminative	PMP - 634	-11.6
*Arnebia euchroma* (Royle) I. M. Johnst. (Boraginaceae)	Havachobe	Root	LHS	Disinfectant	PMP - 211	-8.1
*Artemisia* sp. (Asteraceae)	Afsantin	Herb	H^a^	Stomach tonic, increasing the appetite	83004	-8.5
*Berberis integerrima* Bunge. (Berberidaceae)	Zereshk- e abi	Fruit	LHS	Tonic, laxative, refrigerant, antiseptic	PMP - 619	88.2 ± 1.7^c^
*Biebersteinia* sp. (Geraniaceae)	Chelledaghi	Root	LHS	Anti-rheumatic	PMP - 215	22.7
*Cerasus avium* (L.) Moench (Rosaceae)	Dom- e gilas	Tail	LHS	Cardiac tonic, diuretic	PMP - 309	28.4
*Chelidonium majus* L. (Papaveraceae)	Mamiran	Root	LHS	Anti-diarrhea, anti-liver disease	PMP - 314	0.0
*Cichorium intybus* L. (Compositae)	Kasni	Root	LHS	Diuretic, analgesic, anti-fever	PMP - 213	13.2
*Colchicum* sp. (Colchicaceae)	Suranjan	Rhizome	LHS	Anti-pain in gout	PMP - 214	19.6
*Commiphora* sp. (Burseraceae)	Moql	Resin	LHS	Anti-rheumatic	PMP - 812	20.8
*Cordia myxa* L. (Boraginaceae)	Sepestan	Fruit	LHS	Mucilage, anti-chest complaints	PMP - 730	-15.9
*Crataegus microphylla* C. Koch (Rosaceae)	Sorkhevalik	Leaf	LHS	Cardiac tonic, diuretic, hypotensive	PMP - 306	80.9 ± 1.3
*Cucurbita pepo* L. (Cucurbitaceae)	Tokhm- e kadu	Seed	LHS	Anti-fever, anti-gastrointestinal parasites	PMP - 729	-31.5
*Cynodon dactylon* (L.) Pers. (Gramineae)	Margh	Rhizome	LHS	Diuretic	PMP - 312	9.6
*Datura stramonium* L. (Solanaceae)	Tature	Leaf	LHS	Anti-asthma	PMP - 728	22.3
*Dorema ammoniacum* Don. (Umbelliferae)	Vasha	Resin	LHS	Anti-gastrointestinal parasites	PMP - 815	14.2
*Echinacea purpurea* (L.) Moench (Asteraceae)	Sarkhargol	Aerial part	H	Anti-inflammatory, immune-stimulant	84159	-50.4
*Echinops* sp. (Compositae)	Shekartighal	Resin	LHS	Demulcent, anti-cough, anti-fever	PMP - 817	2.1
*Elettaria cardamomum* (L.) Maton (Zingiberaceae)	Hel- e sabz	Fruit	LHS	Spice, stomach tonic, carminative	PMP - 630	-25.7
*Entada rheedii* Spreng. (Leguminosae)	Qorc- e kamar	Seed	LHS	Ointment for backache	PMP - 724	17.3
*Eugenia caryophyllata* Thunb. (Myrtaceae)	Mikhak	Flower	LHS	Anti-dental pain, disinfectant	PMP - 504	-34.4
*Ferula asssa-foetida* L. (Umbelliferae)	Anqoze	Resin	LHS	Anti-gastrointestinal parasites	PMP - 816	-40.3
*Ferula gumosa* Boiss ***.*** (Umbelliferae)	Barije	Resin	LHS	Stomach tonic	PMP - 814	-36.7
*Foeniculum vulgare* Mill. (Umbelliferae)	Razyane	Seed	LHS	Tonic, diuretic, carminative	PMP - 632	-9.2
*Heracleum persicum* Desf. (Umbelliferae)	Golpar	Fruit	LHS	Spice, disinfectant, carminative	PMP - 631	4.7
*Iris* sp. (Iridaceae)	Rishe- e irisa	Root	LHS	Diuretic, cathartic	PMP - 212	0.0
*Lepidium sativum* L. (Cruciferae)	Tokhm- e shahi	Seed	LHS	Tonic	PMP - 722	-2.2
*Malva sylvestris* L. (Malvaceae)	Panirak	Flower	H	Mucilage, anti-cough	84450	21.7
*Matricaria inodora* L. (Asteraceae)	Babuneh	Flower	H	Carminative, febrifuge	83452	-7.5
*Nymphaea alba* L. (Nymphaeaceae)	Gol- e nilufar	Flower	LHS	Diuretic, sedative	PMP - 501	66.3 ± 1.2
*Onopordon acanthium* L. (Asteraceae)	Khajebashi	Seed	LHS	Hypotensive, diuretic, cardiac tonic	PMP - 714	80.2 ± 2.0
*Passiflora caerulea* L. (Passifloraceae)	Gol- e saati	Flower	H	Spinal depressant, respiratory stimulant	84568	-33.2
*Plantago ovata* Phil. (Plantaginaceae)	Esfarze	Seed	LHS	Laxative, anti-hemorrhoids	PMP - 731	-24.2
*Quercus infectoria* G.Olivier. (Fagaceae)	Jaft	Bark	LHS	Anti-diarrhea, astringent, antibacterial	PMP - 621	93.9 ± 2.5
*Rosmarinus officinalis* L. (Lamiaceae)	Rosaemary	Leaf	H	Spice, herb, carminative, GI irritant	83636	0.0
*Rubus* sp. (Rosaceae)	Tameshk	Leaf	N^a^	Hypotensive, anti-diarrhea	PMP - 404	51.3 ± 1.0
*Salvia macrosiphon* Boiss. (Labiatae)	Tokhm- e marv	Seed	LHS	Demulcent, anti-cough	PMP - 721	-11.7
*Saponaria officinalis* L. (Caryophyllaceae)	Gol- e sabooni	Flower	H	Expectorant, laxative	84680	-33.6
*Satureja hortensis* L. (Labiatae)	Tokhm- e marze	Seed	LHS	Anti-muscle pain, anti-rheumatic	PMP - 732	34.4
*Tanacetum Balsamita* L. (Compositae)	Shahesparam	Leaf	LHS	Stomach tonic, anti-nausea	PMP - 405	-15.2
*Terminalia chebula* Retz. (Combretaceae)	Halil- e zard	Fruit	LHS	Stomach tonic, anti-diarrhea	PMP - 633	17.9
*Terminalia chebula* Retz. (Combretaceae)	Halil- e siah	Fruit	LHS	Cathartic	PMP - 629	15.2
*Teucrium polium* L. (Labiatae)	Maryam nokhodi	Aerial part	LHS	Cold treatment, anti-fever	PMP - 313	2.1
*Vitex agnus-castus* L. (Verbenaceae)	Panj anghosht	Flower	H	Anti-androgen, carminative	84802	31.4
*Zataria multiflora* Boiss. (Labiatae)	Avishan- e shirazi	Leaf	LHS	Cold treatment, carminative	PMP - 310	24.8
*Zea mays* L. (Gramineae)	Kakol- e zhorrat	Herb	LHS	Diuretic, urethra antiseptic	PMP - 311	-35.7

*All medicinal plants were collected in June 2011 except *Rubus* sp. which is collected in June 2012.

^a^LHS, Local Herbal Store; H, Herburatum of Tehran University of Medical Sciences (http://pharmacy.tums.ac.ir); N, North of Iran (Mazandaran province).

^b^PMP, Popular Medicinal Plant.

^c^Values are means ± SD of three measurements for 6 active plants.

**Table 2:** The active medicinal plants with more than 50% ACE inhibition activity at 330 μg/ml concentration

**Scientific name**	**Percent of inhibition**^**a**^
*B. integerrima*	88.2 ± 1.7
*C. microphylla*	80.9 ± 1.3
*N. alba*	66.3 ± 1.2
*O. acanthium*	80.2 ± 2.0
*Q. infectoria*	93.9 ± 2.5
*Rubus* sp.	51.3 ± 1.0

^a^Values are means ± SD of three measurements.

### Tannin test

Out of six active extracts investigated in this study, only the extract of *Q. infectoria* produced a positive gelatin salt block test (Table 3). The presence of greenish-black or bluish-black color after the addition of ferric chloride with negative gelatin salt block test indicated no tannins in other extracts.

**Table 3:** Results of gelatin salt block test in order to detect tannin in extracts

**Scientific name**	**1% Gelatin**	**1% gelatin + 10% NaCl**	**Ferric chloride**
*B. integerrima*	NP^a^	NP	Green/black
*C. microphylla*	NP	NP	Green/black
*N. alba*	NP	NP	Blue/black
*O. acanthium*	NP	NP	Green/black
*Q. infectoria*	P^b^	P	Green/black
*Rubus* sp.	NP	NP	Blue/black

^a^NP, no precipitate; ^b^P, precipitate.

### Total phenolic content and radical scavenging activity

Oxidative stress plays a critical role in cardiovascular disease [15]. Phenolic compounds are responsible for antioxidant activity due to their ability for scavenging free radicals. Therefore, total phenolic content and radical scavenging activities of these six medicinal plants were determined (Table 4). Most of these six extracts had a large phenolic content. In DPPH radical scavenging assay, IC_50_ values of six plant extracts were determined and compared with those of BHT and Trolox as the positive controls (Table 4). *Q. infectoria* possessed more antioxidant activity (IC_50_ value of 1.7 ± 0.03 μg/ml), compared to that of BHT (IC_50_ value of 10.3 ± 0.15 μg/ml) and Trolox (IC_50_ value of 3.2 ± 0.06 μg/ml).

**Table 4:** Total phenolic content and antioxidant activity of active extracts

**Scientific name**	**Tp**^**a**^**(mg gallic acid/100 g dry plant)**	**IC**_**50**_^**b**^**(μg/ml)**
*B. integerrima*	2250 ± 37	15.3 ± 0.20
*C. microphylla*	2000 ± 29	13.1 ± 0.20
*N. alba*	3860 ± 36	10.2 ± 0.15
*O. acanthium*	2740 ± 26	7.0 ± 0.09
*Q. infectoria*	7410 ± 101	1.7 ± 0.03
*Rubus* sp.	3870 ± 26	11.3 ± 0.22
BHT	_	10.3 ± 0.15
Trolox	_	3.2 ± 0.06

^a^Tp, Total phenolic content.

^b^IC_50_, radical scavenging activity in DPPH assay.

Values are means ± SD of three independent measurements.

## Discussion

Hypertension, a worldwide illness, is a major factor in cardiovascular diseases that affects a large population of adults. One of the most effective medications for the treatment of hypertension is angiotensin converting enzyme inhibitors. Meanwhile, medicinal plants have been used for treating illnesses. Therefore, they can be important resources to develop new drug candidates [16].

As illustrated in Table 1 six extracts showed inhibition activity more than 50%, nineteen extracts showed inhibition activity less than 50% and 25 of them showed no inhibition activity at 330 μg/ml concentration. Among these active medicinal plants, three ones (*C. microphylla*, *O. acanthium* and *Rubus* sp.) are utilized for the treatment of hypertension in traditional medicine and this research revealed that the mechanism of action of the three mentioned plants in treatment of hypertension could be done through ACE inhibition.

In addition, Antihypertensive activity is reported in traditional medicine for similar species of the above mentioned plants including *Crataegus oxyacantha*[17], *Onopordon leptolepis* and *Onopordon carmanicum*[18].

Flavonoids [19], flavanols [20], flavonols [21], anthocyanins [22], isoflavones [23], flavones [24] and other phenolic compounds have proved to be effective in decreasing the ACE activity. Therefore, the above mentioned secondary metabolites could be responsible for ACE inhibition activity in these 6 active extracts.

On the other hand, such ACE inhibitors with antioxidant activity might be useful to treat other diseases which are caused as a result of a rise in ROS production [25].

The highest ACE inhibition activity (93.9 ± 2.5%) was observed at 330 μg/ml concentration in *Q. infectoria*. It is clear that tannins are phenolic natural products that interfere in enzymatic activity. Consequently, we checked for its presence in our active extracts (extracts with ≥ 50% ACE inhibition activity). Unfortunately, greenish-black color after the addition of ferric chloride with positive gelatin salt block test indicated the presence of condensed tannins in *Q. infectoria* extract. Therefore, in spite of high ACE inhibition and antioxidant activity, it is not the plant of choice for further studies to isolate the active compounds.

Among other active medicinal plants, three ones including *B. integerrima, C. microphylla* and *O. acanthium* possessed > 80% ACE inhibition activity. Out of the three mentioned plants, *O. acanthium* possessed more antioxidant activity (IC_50_ value of 7 ± 0.09 μg/ml), compared to that of *B. integerrima* (IC_50_ value of 15.3 ± 0.2 μg/ml) and *C. microphylla* (IC_50_ value of 13.1 ± 0.2 μg/ml). Therefore, because of high ACE inhibition and antioxidant activity, *O. acanthium* is a valuable medicinal plant for isolation of active compounds.

Furthermore, *N. alba* and *Rubus* sp. are moderate ACE inhibitors and after the above three mentioned plants, are valuable plants for isolation of active compounds in further studies.

## Conclusions

**Figure 1:**
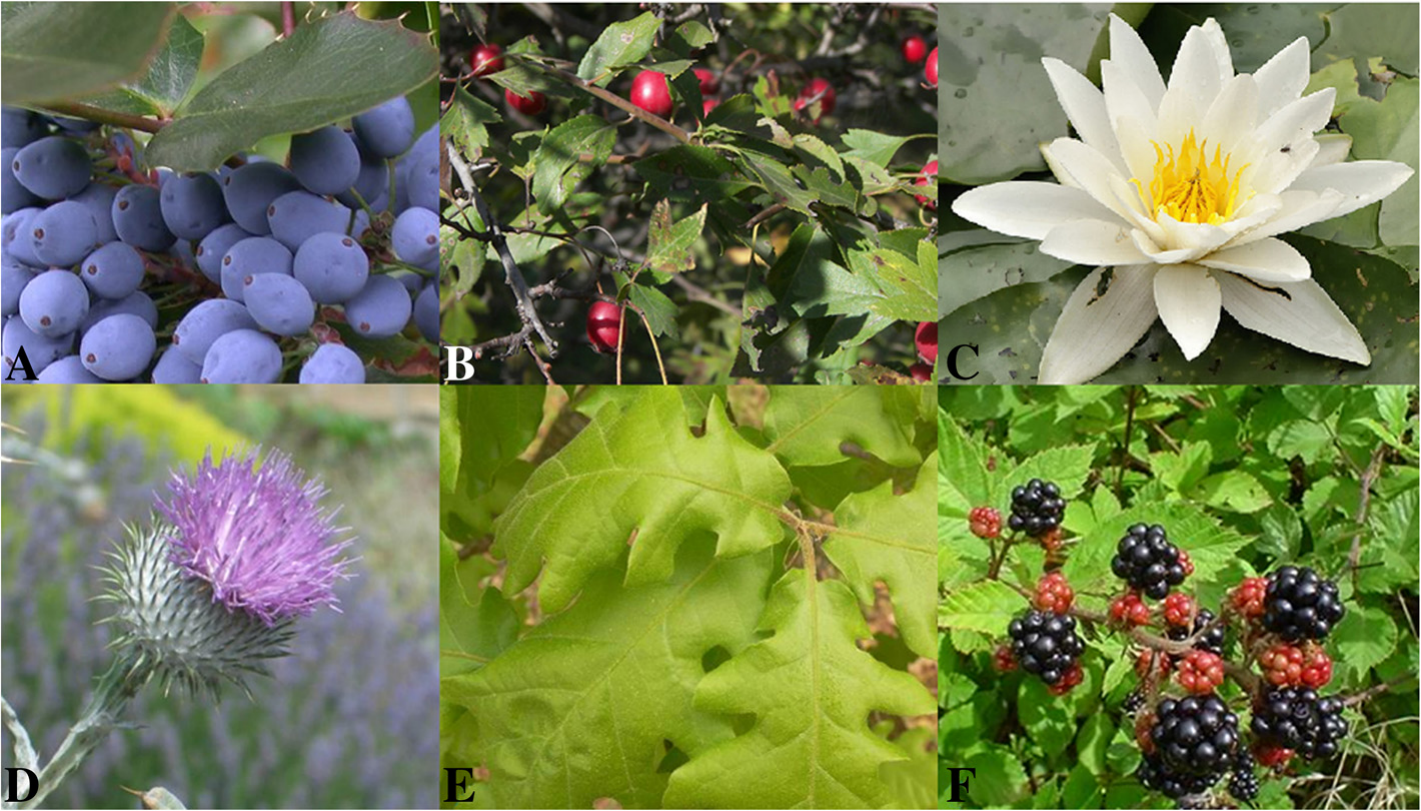
**The picture of active medicinal plants with more than 50% angiotensin converting enzyme inhibitory activity. A)***B. integerrima***B)***C. microphylla***C)***N. alba***D)***O. acanthium***E)***Q. infectoria***F)***Rubus* sp [26-31].

**Figure 2:**
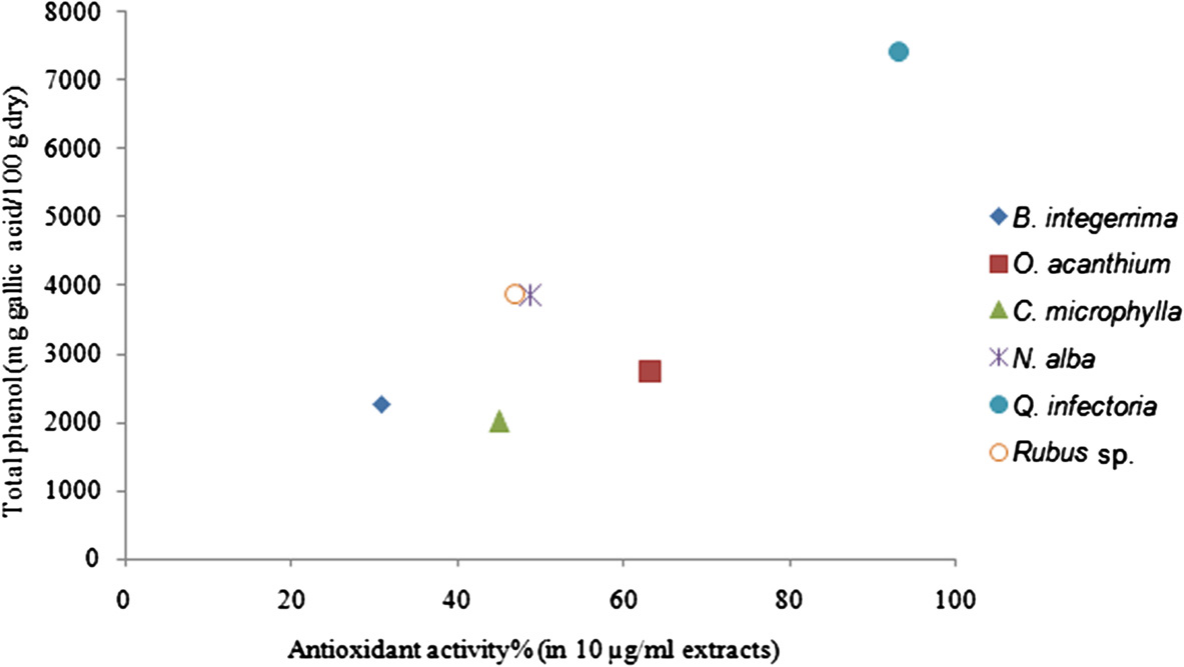
The correlation between total phenolic content and DPPH radical scavenging activity for six plant extracts.

Medicinal plants presented in Table 2 are potential sources for developing new ACE inhibitors (Figure 1) [26-31]. There was a linear relationship between total phenolic content and radical scavenging activity with equation formula, y = 83.53 × - 823.4; R^2^ = 0.714 (Figure 2). As shown in Tables 2, 3 and 4, in spite of possessing the highest ACE inhibition and antioxidant activity, *Q. infectoria* had tannin content. On the other hand, *O. acanthium*, which also had high ACE inhibition and antioxidant activity containing no tannin, and could be used in further studies for isolation of active compounds.

## Competing interests

The authors have no conflict of interests to declare.

## Authors’ contributions

NS: Performed the experimental work including plant extraction, biological tests, data interpretation, and manuscript preparation. ES: Was responsible for HPLC analysis. SAZ: Was involved in designing the experiments. GHA: Identified all plants. MA: Was responsible for the study registration, financial and administrative support, and also gave final approval of the version to be published. All authors read and approved the final manuscript.
